# SPP1 is associated with adverse prognosis and predicts immunotherapy efficacy in penile cancer

**DOI:** 10.1186/s40246-023-00558-5

**Published:** 2023-12-19

**Authors:** Yuantao Zou, Xingliang Tan, Gangjun Yuan, Yi Tang, Yanjun Wang, Cong Yang, Sihao Luo, Zhiming Wu, Kai Yao

**Affiliations:** 1 Department of Urology, Sun Yat-sen University Cancer Center, Guangzhou, 510060 China; 2 State Key Laboratory of Oncology in Southern China, Guangzhou, 510060 China; 3 Collaborative Innovation Center of Cancer Medicine, Guangzhou, 510060 China; 4 State Key Laboratory of Oncology in South China, Guangdong Provincial Clinical Research Center for Cancer, Guangzhou, 510060 China; 5 Department of Urology Oncological Surgery, Chongqing University Cancer Hospital, Chongqing, 400030 China; 6 Chongqing Key Laboratory of Translational Research for Cancer Metastasis and Individualized Treatment, Chongqing University Cancer Hospital, Chongqing, 400030 China

**Keywords:** SPP1 gene, Squamous cell carcinoma of the penis (PSCC), Prognosis, Immune microenvironment

## Abstract

**Background:**

The effect of SPP1 in squamous cell carcinoma of the penis (PSCC) remained unknown. We attempted to clarify the function of the SPP1 gene in PSCC.

**Method:**

Eight paired penile cancer specimens (including penile cancer tissue, paracancerous tissue, and positive lymph node tissue) subjected to whole transcriptome sequencing were analysed to identify differentially expressed genes. We used immunohistochemistry to detect the expression of SPP1 protein and immune cell related proteins in penile cancer tissue. Then, we performed weighted gene coexpression network analysis (WGCNA) to identify the genes related to SPP1 in penile cancer tissue and positive lymph node tissue. Based on the GSE57955 dataset, the CIBERSORT and ssGSEA algorithms were carried out to investigate the immune environment of PSCC. GSVA analysis was conducted to identify the signaling pathways related to SPP1 subgroups. Enzyme-linked immunosorbent assay (ELISA) method was adopted to detect SPP1 level in the serum of 60 patients with penile cancer.

**Results:**

Differential analysis indicated that SPP1 was the most differentially upregulated gene in both penile cancer tissues and positive lymph node tissues. Survival analysis suggested that the prognosis of the low-SPP1 group was significantly poorer than that of the high-SPP1 group. Subsequently, immune-related bioinformatics showed that SPP1 was significantly associated with B cells, CD8 + T cells, CD4 + T cells, macrophages, helper T cells, neutrophils and dendritic cells. The immunohistochemical results showed that the high-SPP1 group was characterized by relatively high expression of CD16 and relatively low expression of CD4. GSVA analysis indicated that high-SPP1 group was significantly associated with immune-related pathways such as PD-L1 expression and the PD-1 checkpoint pathway in cancer and the TNF signaling pathway. ELISA demonstrated that the serum level of SPP1 in patients with positive lymph node metastasis of penile cancer was significantly higher than that in patients with negative lymph node metastasis of penile cancer.

**Conclusion:**

Our study shows that the SPP1 gene might be an effective biomarker for predicting the prognosis and the efficacy of immunotherapy in PSCC patients.

**Supplementary Information:**

The online version contains supplementary material available at 10.1186/s40246-023-00558-5.

## Introduction

Squamous cell carcinoma of the penis (PSCC) is a rare malignancy in developed countries. However, in developing countries such as Africa, PSCC has a high prevalence, which is significantly associated with morbidity and mortality [1, 2].Among all the pathological types of penile cancer, penile squamous cell carcinoma accounts for the majority of cases. Approximately 30~50% of all cases of PSCC are infected by human papillomavirus, which is the known etiology for PSCC and cervix cancer [3–5]. The standard treatment approaches for PSCC are surgery and platinum-based chemotherapy [6–8]. However, some patients will have disease progression or relapse after standard treatment, and there are very limited treatment options for them, so they usually have poor survival [9, 10]. Therefore, it is very important to identify molecular mechanisms associated with PSCC’s recurrent alterations [11, 12]. In recent years, the use of immune checkpoint inhibitors such as anti-CTLA-4 antibodies [13] and anti-PD-1 antibodies [14] has represented a significant paradigm shift for advanced cancers across many oncology types. However, much is unknown about the status of the penile cancer immune microenvironment, which may hinder the application of immunotherapy in penile cancer. Therefore, there is an urgent need to develop a robust biomarker to distinguish different immune patterns of penile cancer.

SPP1 has been reported to be a key gene in many cancers [15–19] and can divide patients into subgroups with different immune cell infiltration or different prognoses. In this study, we aimed to investigate the predictive role of SPP1 and explore whether SPP1 is involved in the regulation of the immune microenvironment of PSCC. Collectively, we intend to provide a new biomarker to predict the outcome of PSCC patients and find suitable treatment strategies for PSCC patients with different immune environments.

## Materials and methods

### Data source

Eight paired PSCC samples (including PSCC, positive lymphatic metastasis [LM] and adjacent normal [N] tissue) were used to conduct whole-transcriptome sequencing analysis. We retrieved 183 paraffin-embedded tumor sections (4-µm thickness) from 183 PSCC patients at Sun Yat-sen University Cancer Center (SYSUCC) between 2000 and 2018. Two pathologists separately determined all specimens’ pathological staging according to the TNM Staging System for Penile Cancer (8th ed., 2017). The Sun Yat-sen University Cancer Center Ethics Committee approved our study (No. 2020-FXY-056), and we acquired informed consent (B2020-073). The GSE57955 microarray dataset (39 PSCC samples) from the Gene Expression Omnibus (GEO) database was included in our study for further analysis.

### Differential expression analysis

To identify biomarkers related to PSCC occurrence and lymphatic metastasis, we conducted differential expression analysis between the two groups in R version 4.2.1. One group was between 8 paired PSCC samples and N, and the other was between positive LM and N. The R package “Limma” was used for analysis. |log2Foldchange|> 2 and *P* value < 0.05 were set as the thresholds for identifying differentially expressed genes (DEGs). We visualized the upregulated and downregulated genes with two volcano plots. Then, Venn diagrams were used to portray the results of intersecting the significant DEGs of the two groups.

### Functional annotation analysis

To investigate the potential functional annotation of target genes, Gene Ontology (GO) analysis with functions including biological pathways (BP), cellular component (CC), molecular function (MF) and Kyoto Encyclopedia of Genes and Genomes (KEGG) pathway analyses were performed through the R package “clusterprofiler.” GO terms with a *P* value < 0.05 and KEGG pathways with a *P* value < 0.05 were considered statistically significant. Then, we conducted gene set enrichment analysis (GSEA) based on two types of data sources. Namely, DEGs between PSCC and N were identified by differential expression analysis, and DEGs between LM and N were identified. We obtained the C2.cp.kegg.symbols.gmt dataset from the Molecular Signatures Database (MsigDB). The pathways with a *P* value < 0.05 were regarded as significantly enriched. Gene set variation analysis (GSVA) was implemented to explore the disparity of biological processes in SPP1-defined patterns based on genesets obtained from the Molecular Signatures Database (MSigDB).

### Immunohistochemistry (IHC)

For IHC, the sliced tissues were later treated with hydrogen peroxide methanol solution, after which a primary antibody was incubated with the tissues at 4 °C overnight. Then, the sections were incubated with horseradish peroxidase conjugated secondary antibody for 30 min at room temperature. We added DAB to detect antibody binding. The sliced tissues were immersed in distilled water once a brown color appeared. We finally adopted the mean values recorded. For each specimen, the total score of SPP1 expression was calculated as staining intensity (0 for no staining, 1 for weak, 2 for moderate and 3 for strong) multiplied by the staining range (1 for 0~25%, 2 for 26~50%, 3 for 51~75% and 4 for 76% above). In addition, we stained CD4 and CD16 for different groups of SPP1 to investigate the content of CD4-positive T cells and NK cells in slice samples.

### Survival analysis

After conducting IHC, we classified all the paraffin-embedded cancer tissues into two groups (high SPP1 expression vs low SPP1 expression) by the score of SPP1 staining. Log-rank *P* < 0.05 was considered significant. The R software “survival” package was used to perform the Kaplan–Meier analysis. To further identify the prognostic value of SPP1, we conducted Kaplan‒Meier survival analysis in some subgroups based on the clinical characteristics of data such as age, T stage and G stage.

### WGCNA

We used the WGCNA package based on R to cluster genes into different modules aiming to construct weighted gene coexpression networks network analysis [20]. Rather than focusing on differentially expressed genes, WGCNA converts the expression matrix into a topological overlap matrix (TOM), using Pearson correlation coefficients to describe the degree of correlation between genes and phenotypes, eliminating the problem caused by multiple hypotheses. We divided all the samples into two subgroups according to the median expression of SPP1. Gene significance (GS) quantified the association between individual genes and the SPP1-defined subgroup. Module membership (MM) represented the correlation between module eigengenes and gene expression profiles. Finally, the correlation between gene modules and traits was calculated to determine the most relevant module, and we chose the modules that were most positively related or negatively related to the corresponding SPP1-defined subgroup.

### Immune-related bioinformatic analysis (CIBERSORT, ssGSEA)

To investigate the immune environment of PSCC, CIBERSORT and ssGSEA algorithms were carried out using R software. CIBERSORT is a deconvolution algorithm to infer cell-type proportions based on data from bulk tumor samples of mixed cell types [21], which include the proportions of 22 types of infiltrating immune cells. The single-sample gene set enrichment analysis (ssGSEA) method was used to quantify the enrichment levels of the 23 immune signatures in each sample by the “GSVA” R package [22].

### SPP1 ELISA

The circulating levels of SPP1 were determined using the corresponding enzyme-linked immunosorbent assay (ELISA) kit (Telenbiotech, TLE083 and TL-E092) according to the manufacturer’s instructions. We obtained the serum of 60 patients with penile cancer from Sun Yat-sen University Cancer Center, of which 20 were patients with negative lymph node metastasis confirmed by postoperative pathology. The other 40 patients had penile cancer accompanied by lymph node metastasis.

### Statistical analysis

All statistical analyses were carried out with R software (version 4.2.1). We divided the dataset into a high-SPP1 group and a low-SPP1 group based on the median immunohistochemical staining score and the median expression level of SPP1. Then, we assessed the statistical significance of differences between the high- and low-SPP1 groups by using Chi-square tests for dichotomous variables. The log-rank test was used in the K‒M survival curve to estimate the survival probability difference between the high-SPP1 expression and low-SPP1 expression groups. Unless otherwise indicated, statistical significance was set at a two-sided P value less than 0.05 for statistical tests.

## Result

### Identification of DEGs and hub genes

**Fig. 1:**
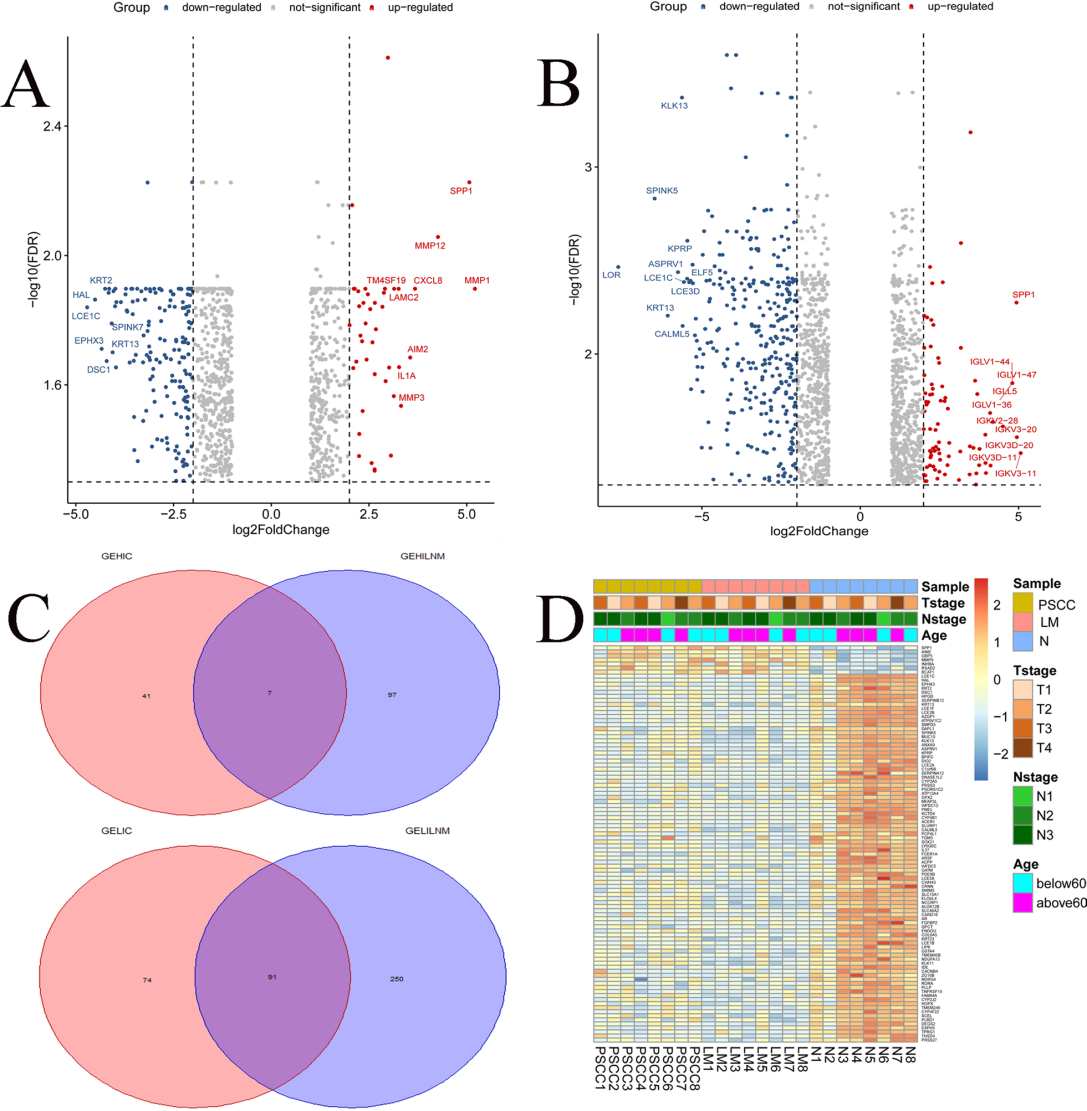
Filtration of genes related to PSCC’s occurrence and lymph node metastasis. **A** Volcano plot exhibiting significantly differentially expressed genes between PSCC and N. **B** Volcano plot exhibiting significantly differentially expressed genes between LM and N. **C** Venn plots revealed 98 intersection genes including 7 upregulated genes in the highly expressed groups and 91 downregulated genes in the lowly expressed groups. **D** Heatmap showing the expression profiles of 98 overlapping genes in 8 paired tissues of penile cancer patients. GEHIC: genes expressed highly in carcinoma of penis, GEHILNM: genes expressed highly in lymphatic node metastasis, GELIC: genes expressed lowly in carcinoma of penis, GELILNM: genes expressed lowly in lymphatic node metastasis, PSCC: squamous cell carcinoma of the penis, LM: lymphatic metastasis, N: normal tissue

Based on RNA-seq data from 8 paired samples, we had three groups of gene expression matrixes. According to the cutoff criteria (P value < 0.05&|log FC > 2|), two columns of DEGs were selected as significant genes. One was making a comparison between PSCC and N, which was shown in Volcano plot 1 (Fig. 1A), and the other was between positive LM and N, which was shown in Volcano plot 2 (Fig. 1B). We made an intersection from two columns of DEGs (Fig. 1C) and obtained 98 genes that were significant in both groups. This is shown in the heatmap (Fig. 1D). Since the 98 genes were significant in all columns, we could hypothesize that the 98 genes had an impact on penile cancer occurrence and metastasis. Among the 98 genes, we found that the SPP1 gene's expression level changed most evidently.

### GO Function analysis

**Fig. 2:**
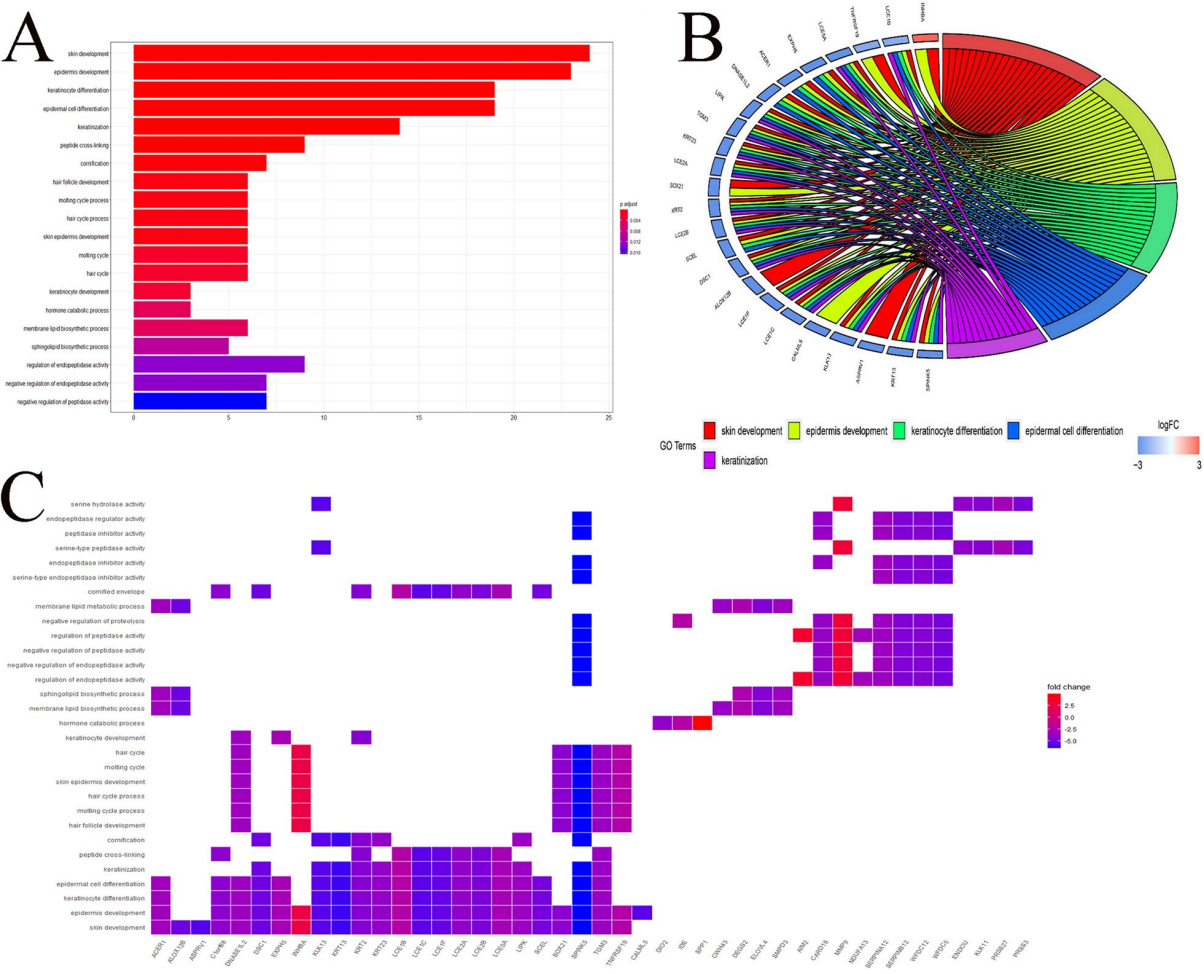
GO analysis based on 98 intersecting genes. **A** Bar plot showing the top 20 biological processes and their corresponding number of genes. **B** Circular plot of the top 5 biological processes and corresponding enriched genes. **C** Heatmap showing the relationship between genes and corresponding biological processes

Gene Ontology (GO) is a database that unifies similar functional genes to predict gene function and trend. According to the results of the GO term functional analysis of 98 DEGs, 98 DEGs associated with penile cancer occurrence and metastasis were mainly enriched in skin development, epidermis development, keratinocyte differentiation, epidermal cell differentiation and keratinization. As shown in Fig. 2A, the 5 functions listed above were mainly regulated by 25 of 98 DEGs. Then, we portrayed the congruent relationship between genes and their function in Fig. 2B and C.

### Gene set enrichment analysis (GSEA)

**Fig. 3:**
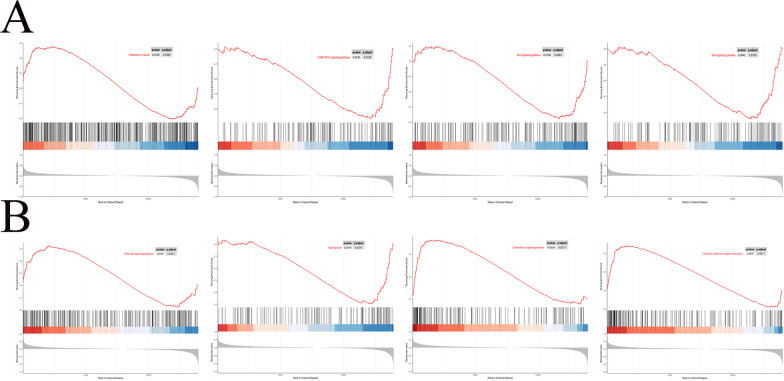
GSEA based on DEGs from the two groups. **A** GSEA based on DEGs derived from comparison between PSCC and N mainly enriched in pathways in cancer, cGMP-PKG signaling pathway, Ras signaling pathway and Wnt signaling pathway. **B** GSEA based on DEGs derived from the comparison between LM and N was mainly enriched in the PI3K-Akt signaling pathway, chemokine signaling pathway, tight junction and cytokine‒cytokine receptor interaction

To understand the underlying molecular mechanisms of the occurrence and metastasis of penile cancer, we performed gene set enrichment analysis (GSEA) based on the DEGs from the two groups. One group was from the comparison between PSCC and N, and the other was acquired from the comparison between LM and N. DEGs from the PSCC group were most significantly enriched for “pathways in cancer,” such as the cGMP-PKG signaling pathway, Ras signaling pathway and Wnt signaling pathway (Fig. 3A). DEGs derived from the LM group were mainly enriched for the “PI3K-Akt signaling pathway” and “chemokine signaling pathway.” In addition, tight junctions and cytokine‒cytokine receptor interactions were also enriched in the LM group (Fig. 3B).

### SPP1 expression in PSCC and some other cancer types

**Fig. 4:**
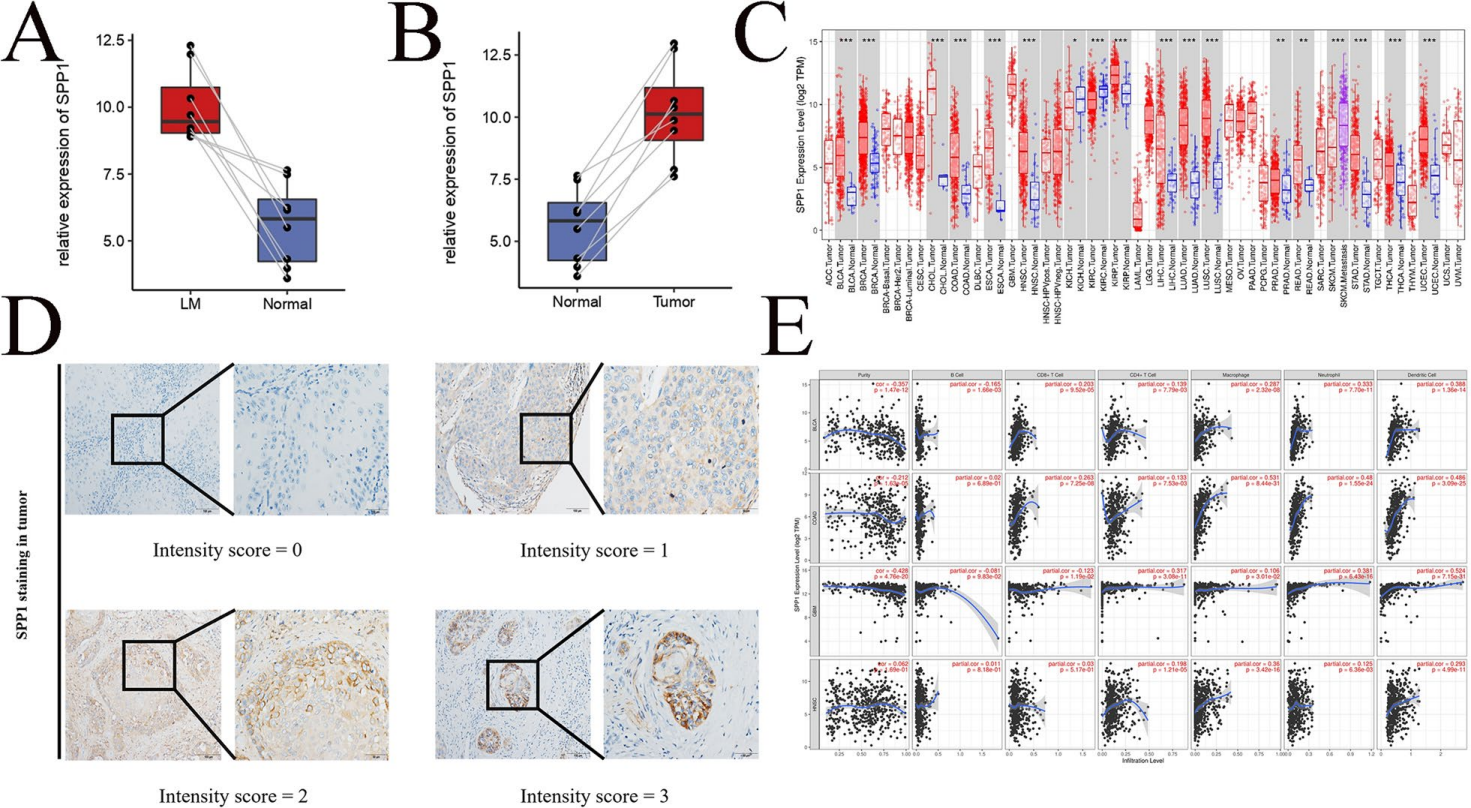
SPP1 expression in PSCC and pan-cancer. **A** Comparison of the expression of SPP1 between PSCC and matched normal tissues in 8 paired tissues. **B** Comparison of SPP1 expression between LM and matched normal tissues in 8 paired tissues. **C** Pan-cancer differential expression of SPP1 was mostly upregulated in cancer tissues compared to normal controls from the TCGA database. *P < 0.05, **P < 0.01, ***P < 0.001 and ****P < 0.0001. **D** Immunohistochemical staining for SPP1 in PSCC tissues. The standard staining intensity score of SPP1 in the cytoplasm was Intensity = 0 for no staining, Intensity = 1 for weak staining, Intensity = 2 for clear staining and Intensity = 3 for strong staining. **E** The SPP1 gene was significantly associated with B cell, CD8 + T cell, CD4 + T cell, macrophage, neutrophil and dendritic cell infiltration in four types of cancers. BLCA = bladder cancer, COAD = colon adenocarcinoma, GBM = glioblastoma, HNSC = head and neck squamous cell carcinoma

For our 8 paired tissues performing RNA-seq analysis, SPP1 was expressed at high levels in PSCC tissue and LM tissue (Fig. 4A, B). To further explore the association between SPP1 protein expression and clinical features, 183 paraffin-embedded PSCC sections were subjected to IHC assays. As detected by IHC, SPP1 proteins were mainly stained in the cytoplasm. Representative immunohistochemical staining pictures are shown in Fig. 4D. The IHC scoring criteria are described in the Materials and methods section, and the frequency in each subgroup is shown in Table 1. In brief, 78 tissues (42.6%) had high expression (IHC score ≥ 2), while 105 (57.4%) had low expression (IHC score ≤ 1). In addition, we found through ELISA that the serum level of SPP1 in patients with penile cancer accompanied by lymph node metastasis was significantly higher than that in patients with penile cancer without lymph node metastasis (Additional file 1: Fig. S1A). Chi-square tests indicated that the high expression of SPP1 was correlated with a lower G stage (*p* = 0.0369). Then, we evaluated the correlation between SPP1 and immune cells in the TIMER database. This shows that the expression of SPP1 was mostly higher in tumors than that in normal tissues (Fig. 4C). In addition, SPP1 was closely related to the infiltration level of immune cells, such as B cells, CD8 + T cells, CD4 + T cells, macrophages, neutrophils and dendritic cells (Fig. 4E).

**Table 1:** Relationship between SPP1 and clinicopathological features in 183 PSCC patients

	High-SPP1 group (*N* = 78)	Low-SPP1 group (*N* = 105)	*P*-value
Age			
< 55	44 (56.4%)	64 (61.0%)	0.641
> 55	34 (43.6%)	41 (39.0%)	
*pT status*			
pT1	34 (43.6%)	26 (24.8%)	0.026
pT2	12 (15.4%)	17 (16.2%)	
pT3	25 (33.3%)	51 (48.6%)	
pT4	6 (7.7%)	11 (10.5%)	
*pN status*			
pN0	44 (56.4%)	49 (46.7%)	0.274
pN1	9 (11.5%)	12 (11.4%)	
pN2	11 (14.1%)	12 (11.4%)	
pN3	14 (17.9%)	32 (30.5%)	
*Metastasis*			
M0	76 (97.4%)	99 (94.3%)	0.506
M1	2 (2.6%)	6 (5.7%)	
*Clinical stage*			
Stage1	24 (30.8%)	22 (21.0%)	0.215
Stage2	21 (26.9%)	24 (22.9%)	
Stage3	16 (20.5%)	23 (21.9%)	
Stage4	17 (21.8%)	36 (34.3%)	
*Histology*			
G1	44 (56.4%)	56 (53.3%)	0.0369
G2	29 (37.2%)	29 (27.6%)	
G3	5 (6.4%)	20 (19.0%)	
*ENE*			
Negative	67 (85.9%)	79 (75.2%)	0.112
Positive	11 (14.1%)	26 (24.8%)	

TNM Staging System and Clinical stage based on the AJCC Cancer Staging Manual for Penile Cancer (8th ed, 2017); PSCC = penile squamous cell carcinoma; ENE = extranodal extension.

### Survival analysis in SPP1-defined subgroups

**Fig. 5:**
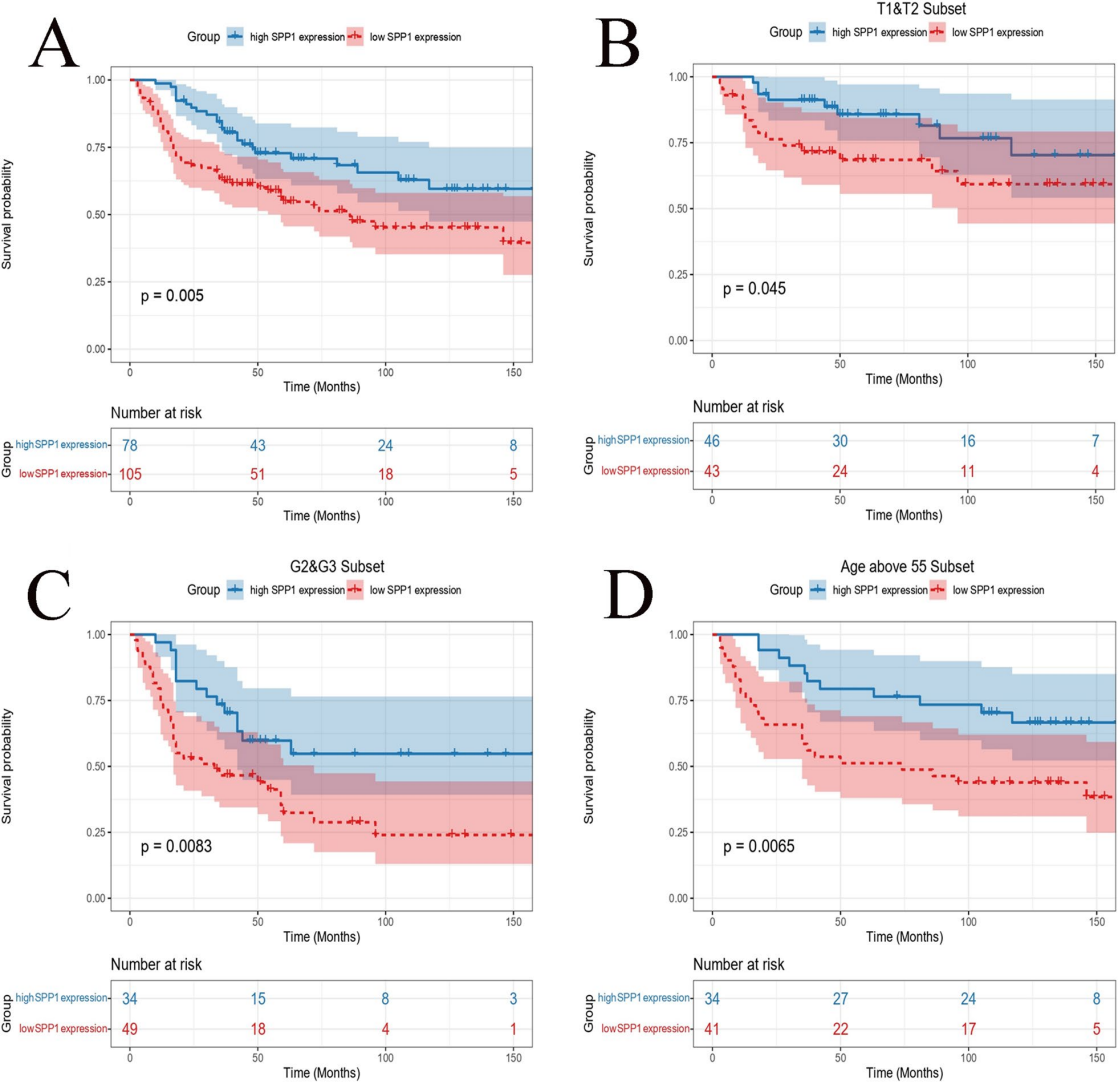
Kaplan‒Meier overall survival of SPP1-defined subgroups. **A** Kaplan‒Meier survival curves indicating overall survival in the high- and low-SPP1 expression groups with PSCC. **B**–**D** Survival curves indicating overall survival in the high- and low-SPP1 expression groups with different clinicopathologic features, including T stage (**B**), G stage (**C**) and age (**D**)

To better explore the effect of SPP1 on PSCC patients’ survival, overall survival was significantly better for patients in the high-SPP1 expression group than for those in the low-SPP1 expression group (*P* = 0.005, Fig. 5A). Then, patients were classified into subgroups according to T stage, G stage and age, and the survival of patients in the high-SPP1 expression group was still higher than that of the low-SPP1 expression group (Fig. 5B–D).

### SPP1-related bioinformatics analysis based on RNA-seq data

**Fig. 6:**
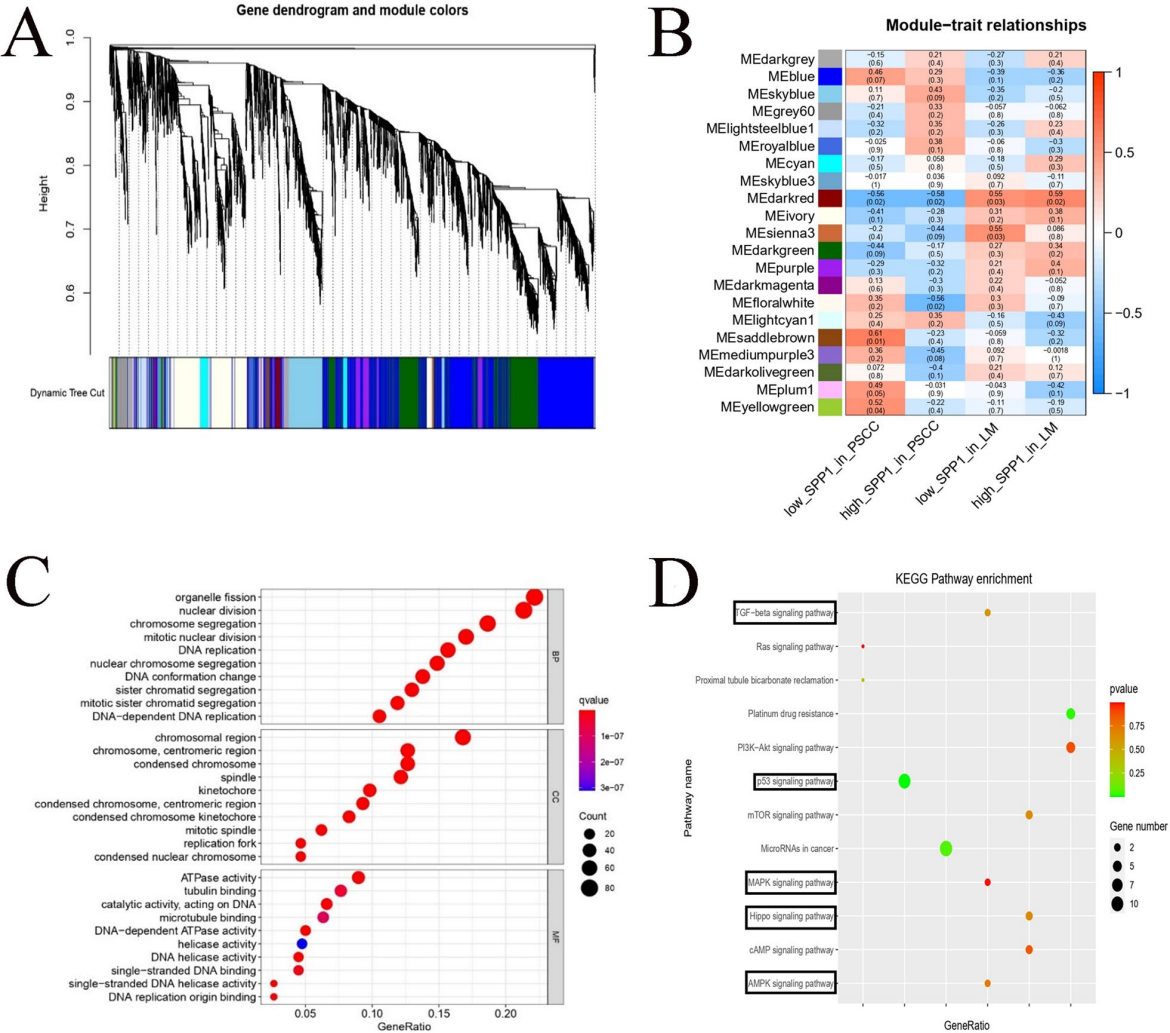
Bioinformatics analysis based on SPP1-related genes. **A** Clustering dendrogram of all different genes clustered based on the measurement of dissimilarity measure (1-TOM) and divided into 21 modules. **B** Heatmap of the relationship between module eigengenes and SPP1-defined subgroups. **C** GO analysis of five module eigengenes. Each rectangular box represents biological processes (BP), cellular components (CC) and molecular functions (MF). **D** Enrichment analysis of Kyoto Encyclopedia of Genes and Genomes (KEGG) for five module eigengenes

In WGCNA, the relationships between all genes and their corresponding modules are shown in a waterfall chart (Fig. 6A). We identified 21 coexpression modules and analyzed their association with SPP1-defined subgroups: the low-SPP1 group in PSCC, high-SPP1 group in PSCC, low-SPP1 group in LM and high-SPP1 group in LM. To select genes tightly associated with SPP1, we chose the modules that were most positively related or negatively related to the corresponding SPP1-defined subgroup. From the results shown in the heatmap (Fig. 6B), we could conclude that the MEdarked, MElightcyan1, MEsaddlebrown, MEsienna3 and MEskyblue modules were mostly related to SPP1. Therefore, these modules were selected for further analysis. GO enrichment analysis suggested that SPP1 was mainly related to the functions of DNA and chromosomes in the nucleus (Fig. 6C). In KEGG pathway analysis, the TGF−beta signaling pathway, p53 signaling pathway, MAPK signaling pathway, Hippo signaling pathway and AMPK signaling pathway were also enriched based on the genes from five modules (Fig. 6D).

### Underlying extrinsic immune landscapes of the high- and low-SPP1 expression groups

**Fig. 7:**
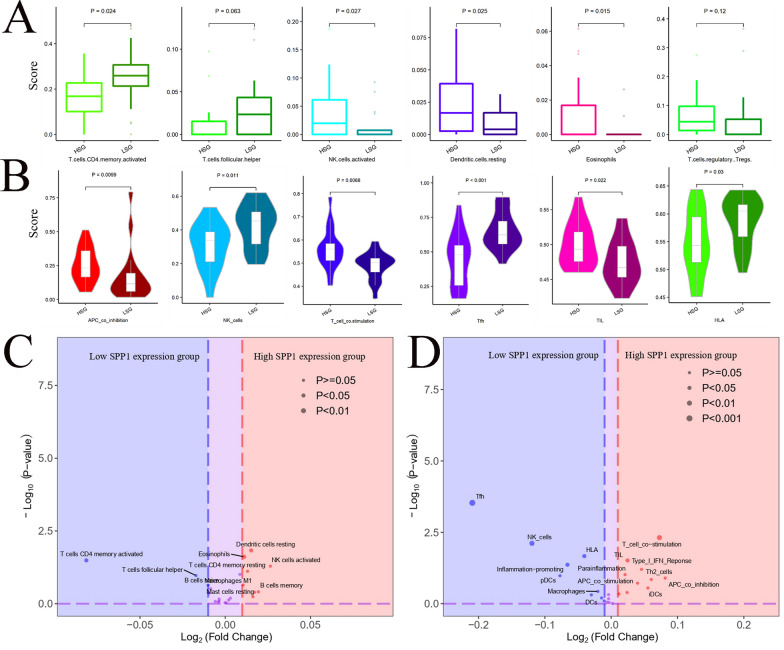
Comparison of immune cell infiltration proportions between the high-SPP1 and low-SPP1 groups. **A** Comparison of immune cell infiltration between the high-SPP1 and low-SPP1 groups by means of CIBERSORT. **B** Comparison of immune cell infiltration between the high-SPP1 and low-SPP1 groups by means of ssGSEA. **C&D** Volcano plots of immune signatures in the high-SPP1 and low-SPP1 groups by CIBERSORT and ssGSEA. Immune signatures enriched in the low-SPP1 group are marked in blue, and immune signatures enriched in the high-SPP1 group are marked in red

To explore the relationship between SPP1 expression and immune cell infiltration, we conducted immune-related bioinformatic analysis, including CIBERSORT and ssGSEA, based on the data from GSE57955. Given that there was a PSCC sample lacking SPP1 expression, we ultimately included 38 specimens for further analysis. The cohort from GSE57955 was classified into high-SPP1 and low-SPP1 groups based on the median value of SPP1. In CIBERSORT analysis, the results showed that a relatively higher proportion of activated NK cells and a lower proportion of activated memory CD4 + T cells were found in the high-SPP1 group than that in the low-SPP1 group (Fig. 7A, C). The results of CIBERSORT analysis were partially consistent with those of immunohistochemistry (Additional file 1: Fig. S1B). To further explore the connection between SPP1 and the immune environment of PSCC, ssGSEA was performed to present the different fractions of immune cell infiltration between the high-SPP1 group and the low-SPP1 group. Enrichment of immune signatures such as APC_co_inhibition and T_cell_costimulation was significantly higher in patients in the high-SPP1 group (Fig. 7B, D).

### Differences between high- and low-SPP1 group-regulated pathways

**Fig. 8:**
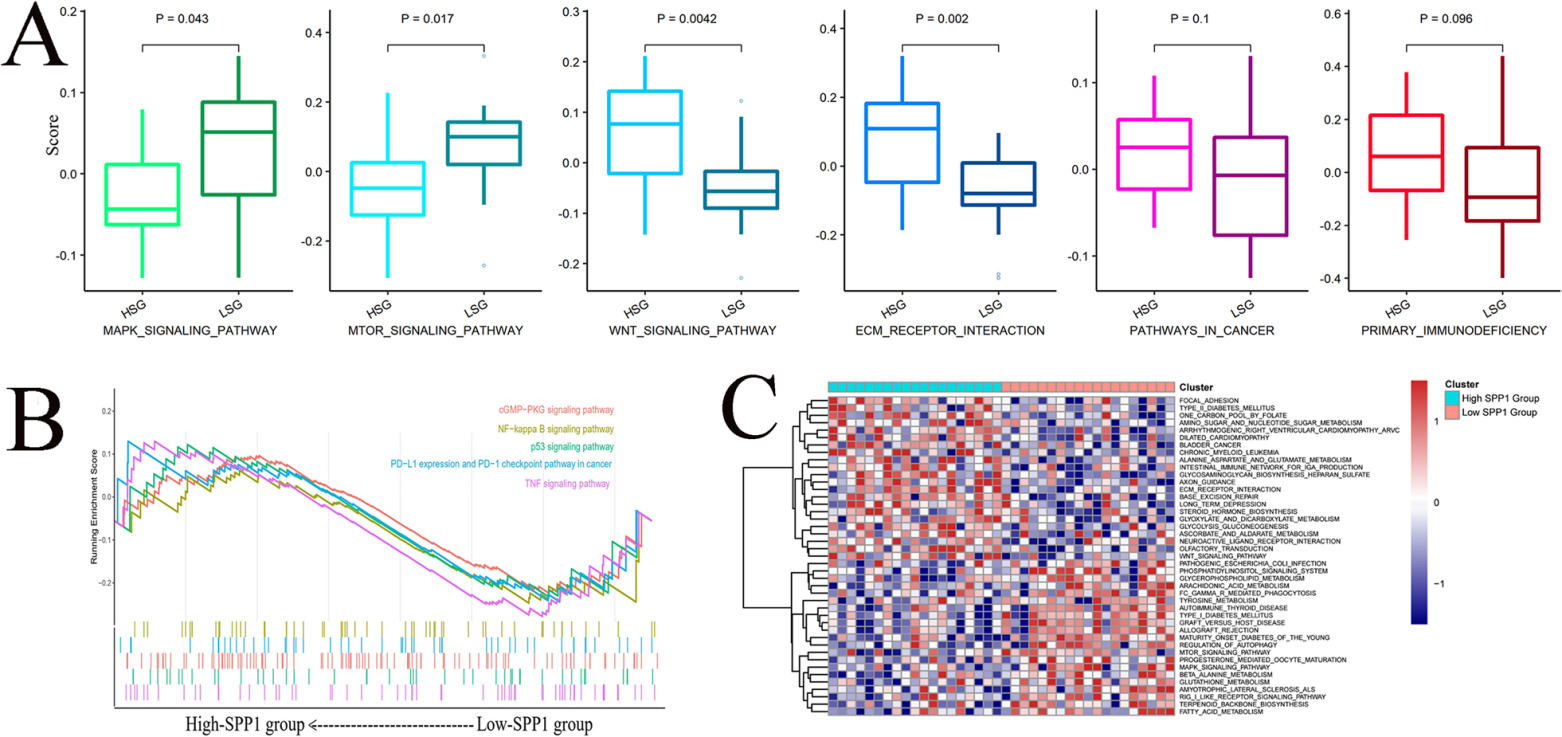
Potential intrinsic immune response and escape landscapes in the high-SPP1 and low-SPP1 groups. **A** Comparison of enrichment scores of oncogenic pathways between the high-SPP1 and low-SPP1 groups. **B** Enrichment plots from gene set enrichment analysis (GSEA) showing differential enrichment of genes in KEGG with high SPP1 expression (including the cGMP-PKG signaling pathway, NF-kappa B signaling pathway, p53 signaling pathway, PD-L1 expression and PD-1 checkpoint pathway in cancer, and the TNF signaling pathway showed significant differential enrichment in the high-SPP1 expression phenotype). **C** Heatmap showing the different GSVA scores of cancer hallmark gene sets between the high-SPP1 group and the low-SPP1 group

The GSVA analysis indicated that signaling pathways such as the WNT signaling pathway and MTOR signaling pathway had highly differential pathway scores, suggesting their positive relationship with the expression level of SPP1 (Fig. 8A). More detailed information about GSVA was presented in Fig. 8C. In addition, the GSEA results suggested that the high-SPP1 group was significantly associated with immune-related pathways, such as PD-L1 expression and the PD-1 checkpoint pathway in cancer and the TNF signaling pathway (Fig. 8B).

## Discussion

Squamous cell carcinoma of the penis is a rare malignant disease associated with low survival rates, limited therapeutic options and increasing recurrence rate, mainly due to the poor understanding of the molecular alterations underlying disease development and progression [3, 23]. There are some typical risk factors, such as phimosis, smoking and HPV infection, that are tightly related to PSCC. However, these high-risk factors do not transform into clinical application in the treatment of PSCC patients [5]. Immunotherapy has become another method for the treatment of malignant tumors after surgery, chemotherapy and radiotherapy and has achieved remarkable curative effects in a variety of solid tumors. However, how to screen patients suitable for immunotherapy is still a problem. Therefore, it is vital to have a good understanding of the molecular alterations involved in PSCC occurrence and progression, which may provide new insights for therapeutics.

Secreted phosphoprotein 1 (SPP1), also known as osteopontin, encodes the protein involved in the attachment of osteoclasts to the mineralized bone matrix. Many studies have reported that abnormal expression of SPP1 is closely related to a variety of tumors [24–26]. However, the clinical significance and biological roles of SPP1 in PSCC have not been elucidated. In this study, we found that the expression of SPP1 is the highest in both PSCC tissue and paired LM tissues, which may suggest that the SPP1 gene plays an important role in the occurrence and metastasis of PSCC. Moreover, the level of SPP1 was significantly associated with clinicopathological parameters, including pathological grade and T stage, which might serve as an independent prognostic factor for overall survival in PSCC. In many previous studies, the SPP1 gene was recognized as an oncogene because its expression level is usually higher in tumor tissue than in normal tissue. A high expression level of SPP1 suggested a poor prognosis of patients. However, the case in the PSCC is quite different. If there is a significant correlation between factor A and factor B, there will be two situations at this time. One is that A regulates B, that is, the increase in A leads to an increase in B. The other case is that the increase in B may lead to an increase in A. The situation illustrated above may also apply to analyze the relationship between tumors and genes. We confirmed that SPP1 was overexpressed in both PSCC tissues and LM tissue; normally, in this case, we would recognize SPP1 as an oncogene. However, in our survival analysis of SPP1 based on IHC data, we found that the high-SPP1 group had a better prognosis than the low-SPP1 group. This revealed a situation that contradicted what we had speculated. Therefore, we proposed a hypothesis that the continuous development of PSCC may lead to increased SPP1 gene expression in the tumor microenvironment. Elevated SPP1 regulates the role of antitumor immune cells and then enhances the antitumor effect.

KEGG analysis based on the genes from WGCNA was applied to discover the pathways that SPP1 might regulate the TGF-β pathway. It is known for its dual effects of promoting and suppressing cancer [27, 28]. This might partly explain the better prognosis of the high-SPP1 expression group. Highly expressed SPP1 has been found to be associated with the phenotype of macrophages [29, 30]. While the effect of SPP1 on the PSCC’s immune environment had not been elucidated, we conducted CIEBRSORT and ssGSEA analyses. We found that the high-SPP1 group featured a stronger pattern of immune activities, such as high levels of T-cell regulatory Treg infiltration, as determined by the CIBERSORT method. When we used the ssGSEA method to calculate the overall immune cell infiltration levels in PSCC, the level of tumor-infiltrating lymphocytes (TILs) was found to be significantly higher in the high-SPP1 group than that in the low-SPP1 group, which again confirmed the elevated antitumor immune activity in the high-SPP1 group. In fact, many studies have shown that the density of TILs is positively related to the immune response in various kinds of tumors [31]. Apart from a high degree of TIL infiltration, the high-SPP1 group was also characterized by higher levels of activated NK cells and resting dendritic cells than the low-SPP1 group. Therefore, activated antitumor immunity and enhanced tumor immunogenicity could explain why high-SPP1 groups are more likely to benefit from antitumor immune activity from the inner immune system than low-SPP1 groups.

Studies have attempted to identify distinct subpopulations and to explore mechanisms underlying disease carcinogenesis and strategies to select patients suitable for immunotherapy [32, 33]. However, studies on penile cancer immunotherapy are still limited due to the lack of cases. Our study suggests that SPP1 gene expression has a tight association with various immune-related cells in the tumor microenvironment of PSCC. Its gene expression may predict the prognosis and immune status of PSCC patients, which makes it a promising biomarker for predicting the efficacy of immunotherapy for penile cancer.

There are several limitations of this retrospective analysis. First, we are lacking in PSCC patients receiving immunotherapy to further verify the reliability of the conclusion. In addition, our data are based on a retrospective analysis, and its reliability needs to be further verified by prospective studies. Despite the strong correlation between the high-SPP1 group and improved tumor immunogenicity as well as inflamed antitumor immunity, it is still necessary to further explore the potential molecular mechanism how SPP1 reacts with immune-related cells.

### Conclusions and expert recommendations

Overall, our bioinformatic analysis identifies SPP1 is the most upregulated gene in PSCC and might have prognostic and predictive value in patients of PSCC. Furthermore, our finding on the immune microenvironment involved in SPP1 gene regulation allows us to make a conclusion that the upregulation of the SPP1 expression in PSCC enhances the immune response mediated by T cell regulatory Tregs and tumor-infiltrating lymphocytes. And the PSCC patients with high SPP1 gene expression usually have a better prognosis than those with low SPP1 gene expression. In conclusion, PSCC subgroups defined by the SPP1 gene have significantly different immune microenvironment and prognosis, which demonstrated that SPP1 might be a reliable prognostic and predictive biomarker of immunotherapy in patients with PSCC.

### Supplementary Information

**Additional file 1. Figure S1A. **Comparison of serum SPP1 levels in penile cancer patients with and without lymph node metastasis by ELISA. **Figure S1B.** CD4 and CD16 staining corresponding to the grouping of high and low SPP1 expression levels.

## Data Availability

Gene expression values based on Gene Expression Omnibus (GEO) are accessible through Series accession number GSE57955. Further inquiries can be directed to the corresponding authors.

## Code availability

Bioinformatics analysis of this study was based on R 4.2.1, and the R packages involved were described in detail in the materials and methods section. All codes were written and reviewed by Zou Y and Yao K. Corresponding author had the ultimate power of interpretation and ownership of the code.
