# Supplementary material for: SPP1 is associated with adverse prognosis and predicts immunotherapy efficacy in penile cancer
Source: Hum Genomics. 2023 Dec 19;17:116. doi: 10.1186/s40246-023-00558-5 (PMC10729401; doi:10.1186/s40246-023-00558-5)
Supplement: Supplementary file 1 — Additional file 1. Figure S1A. Comparison of serum SPP1 levels in penile cancer patients with and without lymph node metastasis by ELISA. Figure S1B. CD4 and CD16 staining corresponding to the grouping of high and low SPP1 expression levels. [file 40246_2023_558_MOESM1_ESM.docx]

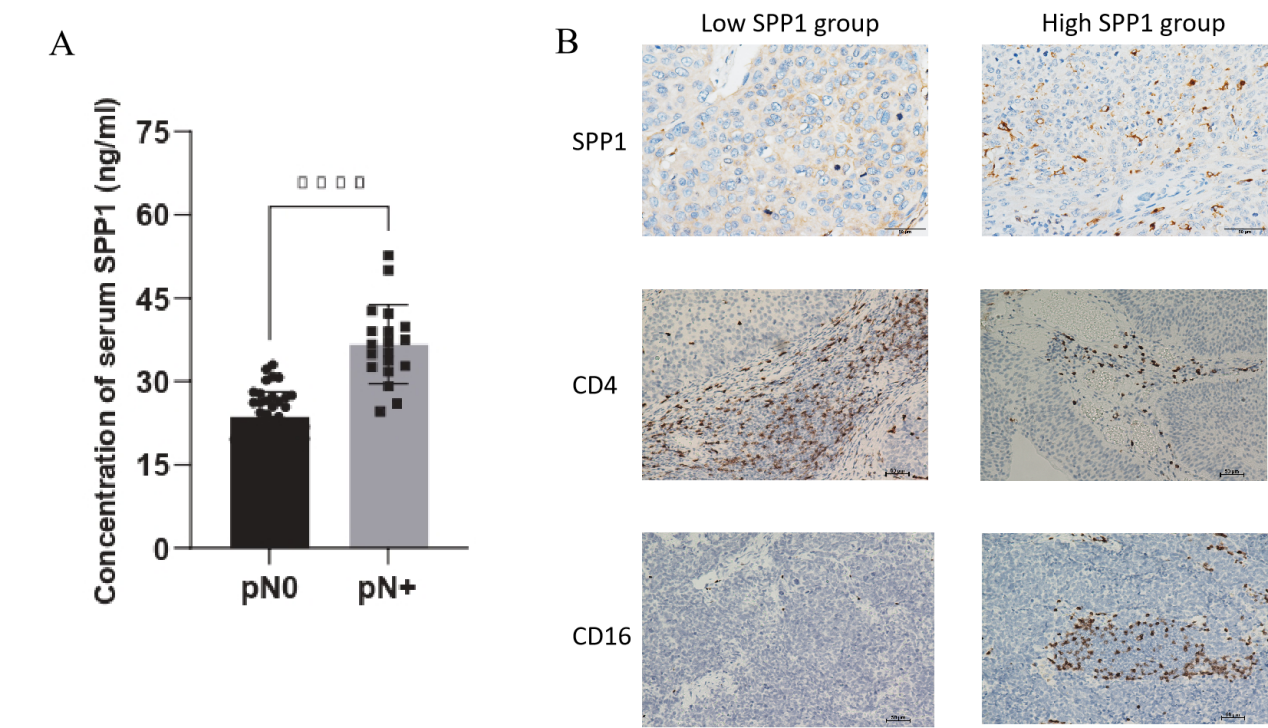


Figure S1 Comparing the characteristics of lymph node grouping and SPP1 expression level grouping

A. Comparison of serum SPP1 levels in penile cancer patients with and without lymph node metastasis by ELISA B. CD4 and CD16 staining corresponding to the grouping of high and low SPP1 expression levels.
